# MRA Mapping and Selective Embolization of a Large Uterine Cavity Pseudoaneurysm at 20 Weeks of Gestation

**DOI:** 10.1155/2018/3610492

**Published:** 2018-05-07

**Authors:** Jean V. Storey, Timothy B. Dinh, Deirdre M. McCullough, Steven H. Craig, Christian L. Carlson

**Affiliations:** ^1^Department of Obstetrics and Gynecology, Brooke Army Medical Center, 3551 Roger Brooke Drive, Fort Sam Houston, San Antonio, TX 78234, USA; ^2^Department of Radiology, Brooke Army Medical Center, 3551 Roger Brooke Drive, Fort Sam Houston, San Antonio, TX 78234, USA

## Abstract

Antepartum uterine cavity pseudoaneurysm rupture can cause massive hemorrhage with high maternal and fetal mortality risk. Invasive placentation can predispose to vascular malformations. We present a novel use of macrocyclic intravenous contrast-enhanced magnetic resonance angiography for preprocedure planning followed by selective low radiation embolization of a uterine cavity pseudoaneurysm in the setting of invasive placentation at 20 weeks of gestation. To our knowledge, this is the first reported case of uterine cavity pseudoaneurysm successfully mapped with MRA and treated with embolization at 20 weeks of gestation.

## 1. Introduction

Uterine surgery can predispose to future pregnancy complications to include abnormal placentation and vascular lesions such as arteriovenous malformations and fistulas and pseudoaneurysms [1, 2]. Hormonal and hemodynamic changes also play a role in development of vascular lesions in pregnancy [3]. Vascular lesions may present with life-threatening hemorrhage. Low patient risk and relatively low cost make ultrasound the screening method of choice with contrast angiography used for problem solving, treatment planning, and therapeutic intervention [1, 4]. Historically, treatment required hysterectomy, but arterial embolization currently offers a less invasive, fertility-sparing treatment option with a low complication rate [1, 5]. We report a novel use of dynamic IV contrast-enhanced magnetic resonance angiography (MRA) for further characterization and treatment planning/mapping of a uterine cavity pseudoaneurysm in a 20-week gravid female followed by low radiation embolization of feeding arteries, resulting in successful maternal and fetal outcome.

## 2. Case Presentation

**Figure 1:**
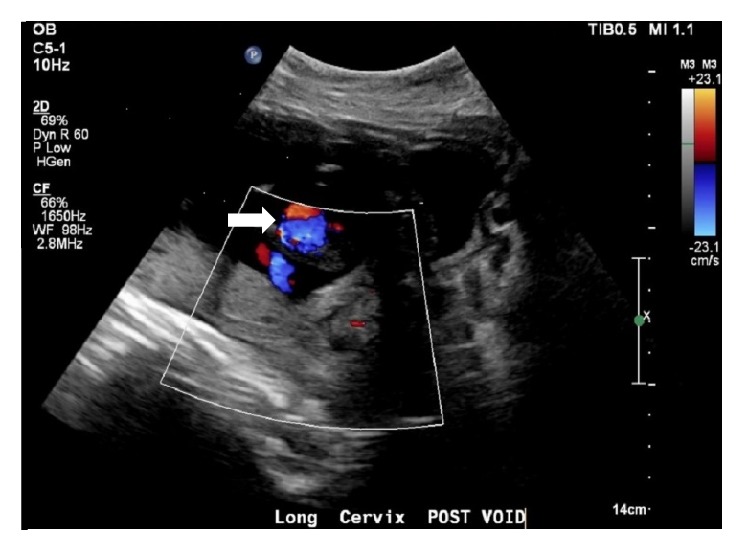
Follow-up ultrasound at 16 weeks and 6 days of gestation reveals a vascular malformation (arrow) at the lower uterine segment with swirling flow within the malformation consistent with a pseudoaneurysm.

**Figure 2:**
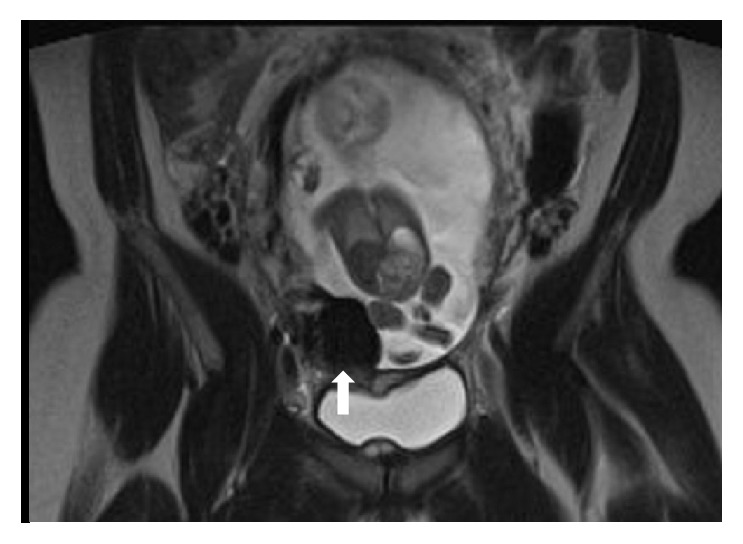
Coronal T2-weighted image of the pelvis at 18 weeks of gestation demonstrates a hypointense round structure (arrow) at the lower uterine segment infringing upon (and invaginating into) the gestational cavity. Fetal knee abuts and deforms the lesion.

**Figure 3:**
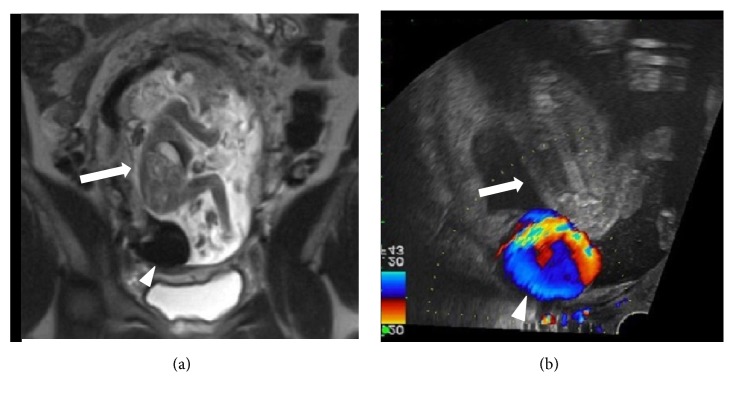
Maternal coronal (sagittal fetal) T2-weighted image of the pelvis (a) at 18 weeks of gestation with similar fetal positioning in relation to the lesion on ultrasound (b) as if the fetus (arrow) is sitting (or bouncing) on the lesion (arrowhead). Note the Yin/Yang swirling flow on color Doppler.

A 35-year-old gravida 4 para 3 with a history of three previous cesarean deliveries presented at 16 weeks 6 days of gestation for follow-up ultrasound of perigestational hemorrhage seen at 10 weeks and 4 days. A large uterine cavity pseudoaneurysm measuring 4.2 × 3.8 × 3.7 cm and appearing to arise from abnormal placentation at the previous cesarean scar was identified (Figure 1). Repeat ultrasound six days later revealed a normal active fetus in breech position compressing the pseudoaneurysm upon contact. An unenhanced MRI one week later confirmed a 4 cm hypointense lesion projecting into the lower right uterine cavity at the inferior margin of the placenta (Figures 2 and 3). Management options were discussed to include conservative imaging observation versus embolization. Due to high maternal mortality risk from spontaneous hemorrhage, elective termination was also discussed but was rejected by the patient.

A novel use of dynamic time-resolved contrast-enhanced MRA utilizing a functional MR urography protocol™ was performed for enhanced characterization of feeding arteries and treatment planning/mapping [6]. The specific MRA parameters utilized can be viewed in detail online at www.chop-fmru.com. Although not FDA-approved for a second trimester fetus, Gadobutrol® contrast agent was selected to reduce risk of gadolinium deposition. Gadobutrol is a macrocyclic agent that imparts strong chelation of the substrate to gadolinium. It reduces potential toxicity from free gadolinium and, at the time, was the only FDA-approved agent for patients below 2 years of age (down to 37 weeks of gestation). Gadobutrol was dosed per the manufacturer's protocol with recommended weight-based dosing of 0.1–0.3 mmol/kg. Informed consent was obtained for this unique use of MRA at 20 weeks of gestation.

**Figure 4:**
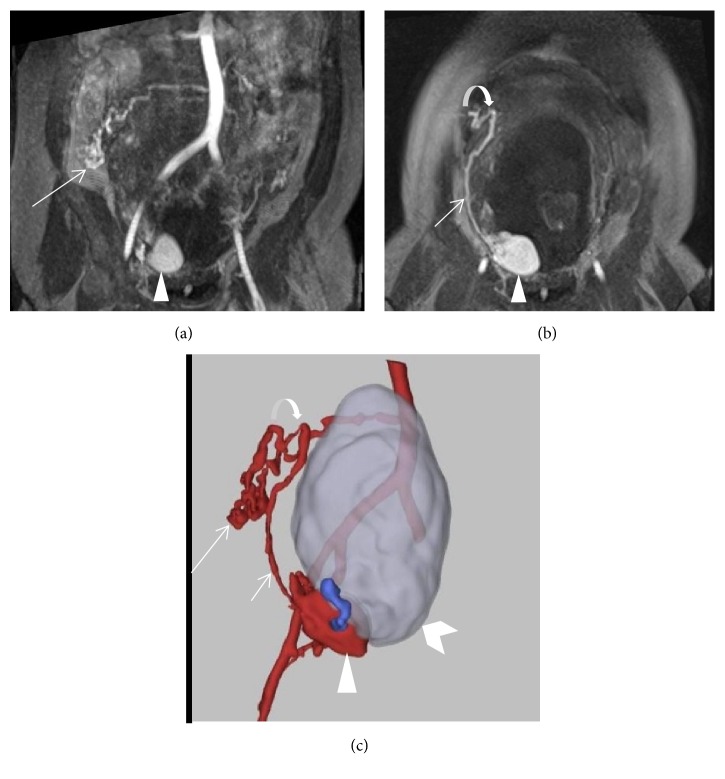
Coronal T1 MRA and 3D surface rendered reconstruction (Vital Images® postprocessing software) of the aberrant right ovarian artery branch feeder at 19 weeks of gestation. The proximal right ovarian artery originates from the aorta and courses into a tangle of vessels (long arrows) in the right abdomen ((a) and (c)) before penetrating the myometrium (curved arrows) ((b) and (c)) and extending caudally (short arrows) as an arcuate artery to the lesion. Pseudoaneurysm (arrowhead), uterine cavity (chevron), and aortoiliac vessels. Draining vein (blue).

**Figure 5:**
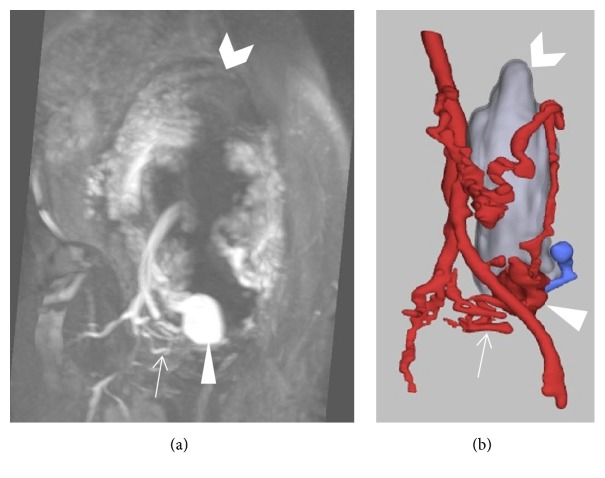
(a) Sagittal oblique reconstruction of T1 MRA and 3D surface rendered image (Vital Images postprocessing software) (b) of uterine artery feeder (arrows) to the pseudoaneurysm (arrowheads). Uterine cavity (chevrons).

MRA revealed two suspected feeding vessels: a branch off the right ovarian artery parasitized to the uterine arcuate artery (Figure 4) and a branch off the right uterine artery parasitized to the uterine arcuate and radial arteries (Figure 5). The lesion now measured nearly 5 cm. Abnormal placentation was again suggested.

**Figure 6:**
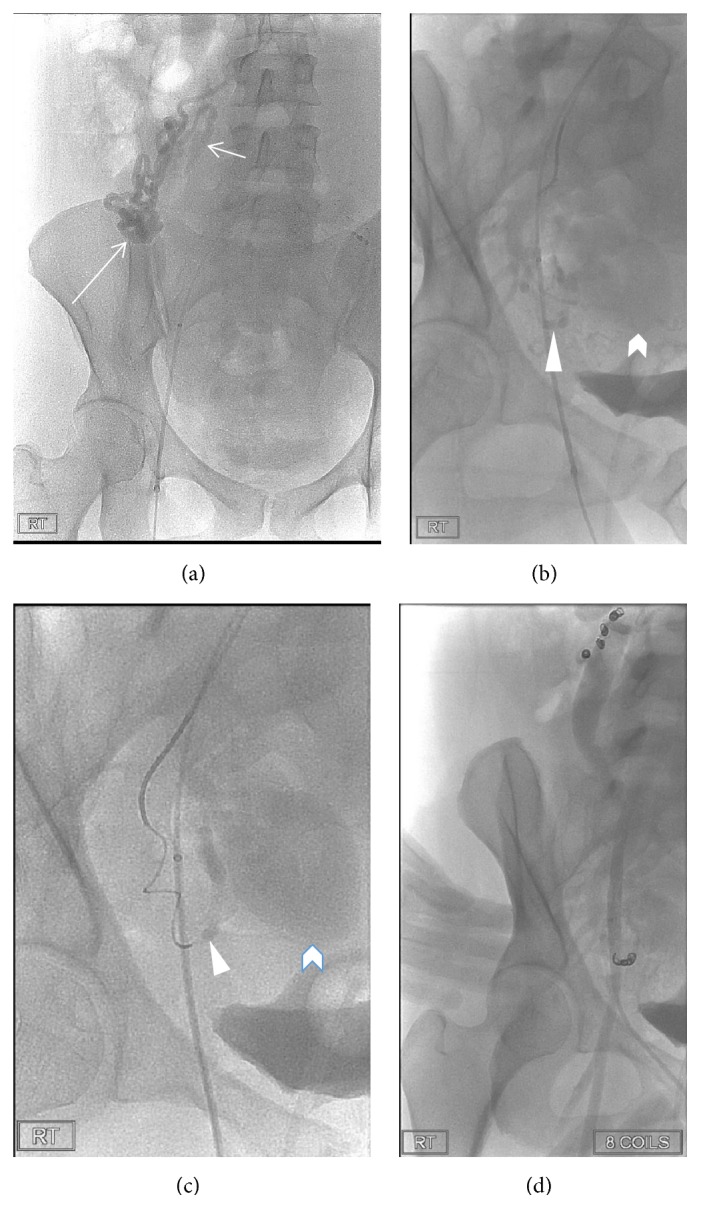
Interventional fluoroscopic images. (a) Tortuous right ovarian artery off the aorta (long arrow) with perforating branch into the myometrium (short arrow). Faint blush of pseudoaneurysm on real-time imaging (not shown). (b) Selected right hypogastric artery showing uterine branch (arrowhead) leading to faint oval blush of pseudoaneurysm (chevron). (c) Subselected uterine artery feeder (arrowhead) and pseudoaneurysm blush (chevron). (d) Postcoiling images of parasitized right ovarian and uterine arteries without flow to the target lesion.

Fetal and maternal risks of embolization were reviewed with the patient who strongly desired intervention. A conventional arteriogram performed with iodinated contrast diluted 50/50 with normal saline demonstrated a prominent right ovarian artery with origin off the aorta at L2/3 as seen on MRA. Prominent hypogastric arteries were noted along with a subtle blush in the right pelvis suspicious for the target lesion. A right ovarian arteriogram revealed a prominent tortuous right ovarian artery similar to that seen on MRA (Figure 6). A more distal right ovarian arteriogram suggested a blush of contrast in the pelvis suggestive of the target lesion. The right ovarian artery was then embolized with coils (Figure 6). A right hypogastric arteriogram revealed a prominent right uterine artery and a large ovoid lesion opacifying with contrast consistent with the target lesion (Figure 6). The right uterine artery was then embolized. Postcoil imaging revealed no lesion opacification (Figure 6). In an effort to reduce radiation dose to the fetus, all angiographic runs were performed without digital subtraction. The required 30.4 minutes of fluoroscopic time resulted in a total radiation dose of only 490 mGy.

**Figure 7:**
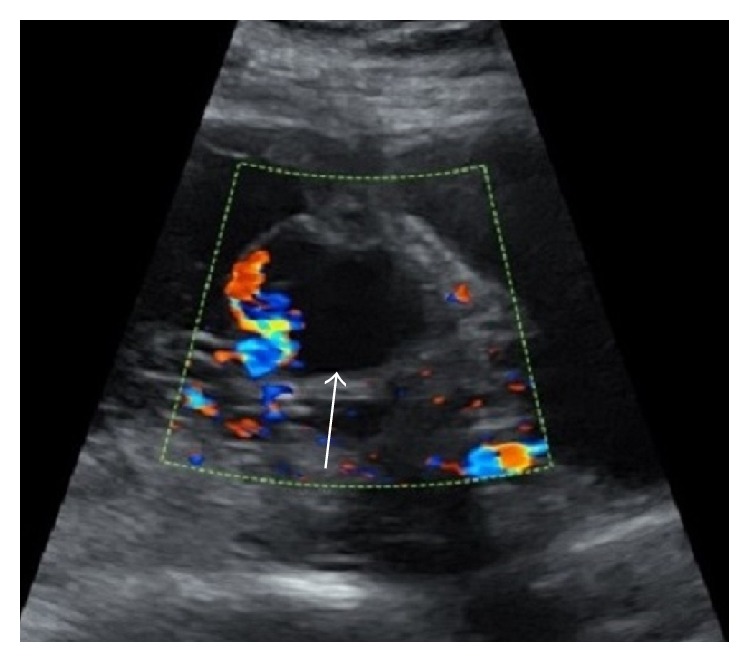
Postprocedure ultrasound the next morning demonstrates significantly reduced flow within the pseudoaneurysm (arrow).

**Figure 8:**
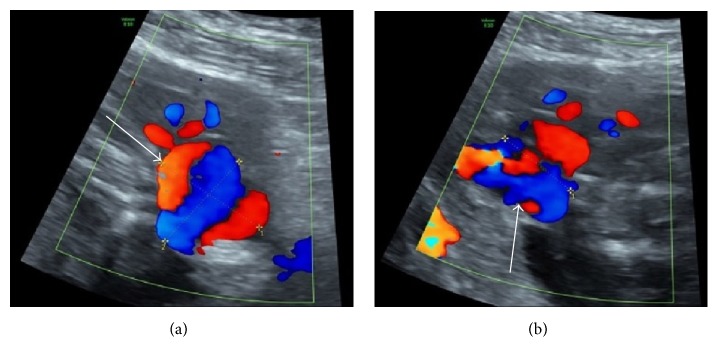
Transverse (a) and longitudinal (b) ultrasound two days after procedure reveal return of flow to the lesion, however, with significant reduction in lesion size to 3.3 cm (arrows) form 5 cm before embolization.

**Figure 9:**
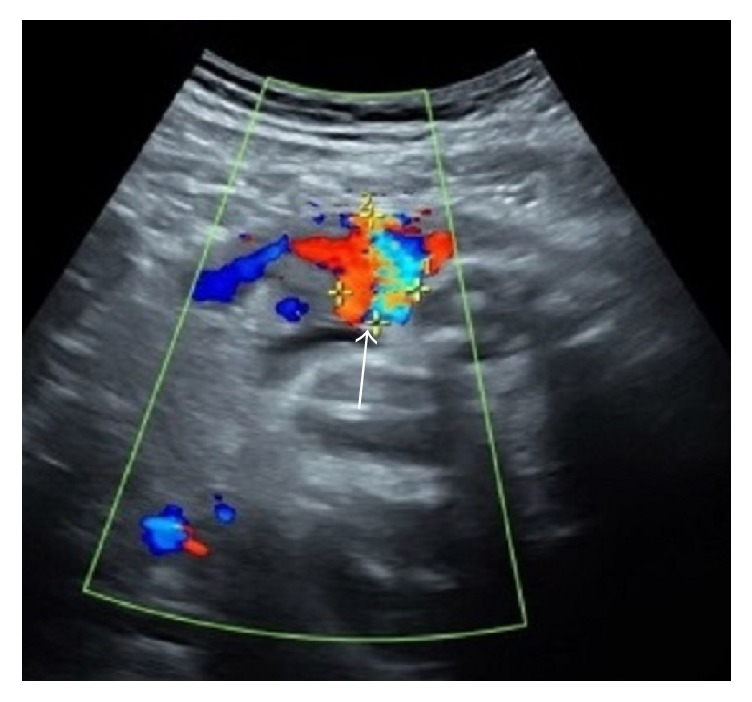
Final ultrasound image taken prior to delivery demonstrates persistence of the aneurysm, although it was significantly reduced in size to 2.0 cm (arrow).

Ultrasound interrogation the next morning revealed no flow within the lesion (Figure 7). Repeat ultrasound 24 hours later, however, showed recurrence of small blood flow into the lesion, with a significant decrease in lesion size to 3.3 cm, which remained stable prior to discharge 4 days later (Figure 8). Serial ultrasound examinations throughout the duration of the pregnancy demonstrated appropriate interval fetal growth. The pseudoaneurysm progressively decreased in size, measuring 1.4 × 2.0 cm just prior to delivery (Figure 9).

The patient presented with premature rupture of membranes at 33 weeks. Cervical changes and painful contractions necessitated an urgent prophylactic cesarean delivery 13 weeks after embolization. On attempt to deliver the placenta, it was adherent to the uterus, consistent with invasive placentation. The placenta was left in situ and a supracervical hysterectomy was performed. The patient was discharged on postoperative day 3. The baby was admitted to the NICU secondary to prematurity and discharged home in stable condition at 19 days of life.

## 3. Discussion

Repeat cesarean delivery increases the risk of abnormal placentation and predisposes to vascular malformations [1, 2]. Hormonal and hemodynamic changes in pregnancy contribute to development of vascular lesions [3]. Pseudoaneurysms develop when trauma, degeneration, or necrosis causes a defect or weakening in the arterial wall through which blood escapes, forming a contained hematoma with or without a thin wall of adventitia and in continuity with the artery that supplies continuous blood flow. Absence of a three-layered arterial wall lining differentiates pseudoaneurysm from true aneurysm [1, 4]. We hypothesize that pseudoaneurysms can occur from invasive processes such as abnormal placentation at the cesarean scar, resulting in abnormal vasculature predisposing to pseudoaneurysm formation.

Research suggests that the initial insult in placenta accreta is a deciduomyometrial defect secondary to surgical scarring. The defect exposes myometrium and its vasculature to migrating trophoblasts, leading to morbidly adherent placenta and loss of the normal cleavage plane between the placenta and myometrium, resulting in excessive remodeling of myometrial arteries [2]. Likely a secondary complication of invasive placentation, our pseudoaneurysm arose at the site of uterine cesarean scar and bulged into the uterine cavity with parasitized, remodeled feeding vessels traversing the myometrium and placenta. The initial perigestational hemorrhage may have been the first signal of abnormal placentation and vascular insult.

Rupture of uterine arterial pseudoaneurysms may cause sudden vaginal hemorrhage unresponsive to typical interventions [1]. Ultrasound is an effective screening modality for pseudoaneurysm and typically reveals an anechoic cyst on grey scale, characteristic “Yin/Yang” or swirling color flow pattern, and bidirectional waveform on duplex Doppler. Most pseudoaneurysms will increase in size over time and eventually rupture with risk of rupture proportional to size and hydrostatic pressure [3]. Pregnancy is a hyperdynamic state, with up to 20% of maternal cardiac output being directed toward the uterus, increasing the risk of pseudoaneurysm formation and rupture [7]. A pseudoaneurysm extending into the intrauterine cavity without a source of tamponade is especially dangerous and hemorrhagic shock can quickly develop with exsanguination into the uterine cavity.

Rebarber et al. reported the first case of bilateral uterine artery embolization at 20 weeks of gestation in 2009 for treatment of an 8 cm lower uterine segment arteriovenous malformation with a successful pregnancy outcome [8]. Ours is the first second trimester case of uterine cavity pseudoaneurysm reported with successful selective unilateral embolization. No immediate postprocedure fetal or maternal complications were encountered. Complete disappearance was noted immediately after the procedure, with partial recurrence observed at 36 hours after embolization. This recurrence was thought to be secondary to the remarkable collateral blood flow of the uterus. Preservation of this collateral blood flow was an important consideration in planning the procedure. The decision was made to proceed with coil rather than particle embolization as particle embolization would have caused more distal embolization, potentially leading to necrosis and vascular compromise of the placenta and poorer fetal outcome.

Lesion overall size significantly decreased from 5.0 cm before the procedure to 2.0 cm just prior to delivery. This progressive decrease in size throughout the remainder of the pregnancy was felt to significantly decrease the risk of rupture and associated complications. At the time of initial recurrence, the benefits of further embolization were considered. However, the potential benefit did not outweigh the risk of additional fetal and maternal exposure to radiation and iodinated contrast. Tighter collimation during the embolization could have reduced the radiation dose further. However, the dose of radiation was very low for this complex procedure and was achieved by performing the procedure without digital subtraction, further decreasing radiation dose.

This case demonstrates that dynamic MRA with judicious use of gadolinium may be safe and can aid in characterization and treatment planning of antepartum uterine cavity vascular lesions without radiation to the fetus. This case also demonstrates that low radiation selective embolization is an appropriate and effective treatment option for high-risk antepartum uterine cavity vascular lesions as an alternative to pregnancy termination and surgical intervention when patients desire to maintain an ongoing pregnancy and/or preserve fertility. Preembolization mapping with MRA may help reduce fluoroscopy time and radiation dose at embolization. Future cases of antepartum uterine cavity vascular lesions may benefit from MRA followed by low dose selective embolization.
